# Tailoring Segmented Polyurethane Foams for Bone Tissue Engineering: Effects of Hard Segment Content and Dual-Function Microhydroxyapatite

**DOI:** 10.3390/ma19194073

**Published:** 2026-09-23

**Authors:** Patrycja Szczepanska, Natalia Dzialecka, Hieronim Szymanowski, Marta Kaminska, Sebastian Lipa, Pawel Petelewicz, Aleksandra Kozlenko, Bartlomiej Januszewicz, Anna Sobczyk-Guzenda

**Affiliations:** Institute of Materials Science and Engineering, Lodz University of Technology, 1/15 Stefanowskiego Str., 90-924 Lodz, Poland

**Keywords:** biodegradable polyurethanes, microhydroxyapatite, solid structure, porous structure, cancellous bone, composites

## Abstract

Poly(ε-caprolactone)-based segmented polyurethanes (PUs) were developed as potential materials for bone tissue engineering, with particular emphasis on porous substitutes for cancellous bone. PUs with hard segment (HS) contents ranging from 17.6 to 32.0% were synthesized from poly(ε-caprolactone) diol, 1,6-hexamethylene diisocyanate, and 1,4-butanediol. The effects of HS content and post-synthesis incubation temperature (40 and 80 °C) on the structural, thermal, surface, mechanical, and biological properties were investigated. The polyurethane containing 25.5% HS exhibited a favorable combination of structural and mechanical properties and was selected for the preparation of porous composites containing microhydroxyapatite (μHAp) and ZrO_2_. The resulting composites exhibited high open porosity (70.4–73.3%). The physicochemical characteristics of μHAp support its proposed role as a predominant water-carrying ceramic phase promoting in situ CO_2_ generation during polyurethane crosslinking; however, because μHAp and ZrO_2_ were incorporated simultaneously, their individual contributions to foaming cannot be unequivocally distinguished. Extract-based MTT testing showed no cytotoxic effects under the investigated conditions. Furthermore, the 30-day stability assessment at 37 °C revealed low mass loss, preservation of Young’s modulus, and only minor changes in molecular weight parameters, supporting the short-term stability of the investigated materials. Overall, the results support further development of these porous PU/ceramic composites as materials for bone tissue engineering.

## 1. Introduction

Tissue engineering (TE) and regenerative medicine (RM) are fields of biomedical engineering that are developing rapidly due to the growing demand for effective tissue repair and regeneration strategies. Tissue engineering is primarily concerned with three-dimensional scaffolds that provide a suitable environment for cells, whereas regenerative medicine aims to regenerate or replace damaged cells, tissues, or organs and restore their normal functions. Bone regeneration involves the restoration of bone tissue with the required shape and function following partial bone loss [1,2]. For many years, TE and RM have sought to address the clinical need for bone repair, and both inorganic and organic bone substitutes have been investigated as alternatives to bone grafts [3,4].

The primary requirement for bone substitutes is their ability to integrate with the surrounding bone while providing adequate mechanical support [3,5]. Current approaches to bone replacement or restoration have several limitations [6]. The functional requirements of a bone-replacement material depend strongly on its intended application. Porous scaffolds and cancellous bone substitutes should provide a three-dimensional structure favorable for tissue ingrowth while maintaining mechanical properties appropriate for the implantation site. In contrast, injectable bone cements intended for applications such as vertebroplasty or kyphoplasty must additionally satisfy application-specific requirements, including appropriate injectability, rheological and handling characteristics, working and setting times, curing behavior, radiopacity, and mechanical compatibility with the surrounding bone. Scaffold-based bone tissue engineering may be insufficient in certain clinical situations, including large bone defects or procedures requiring stabilization and filling of irregular bone cavities. For these reasons, bone cements remain an important group of biomaterials used in bone surgery [3,7,8]. Bone cements are a group of biomaterials that are obtained by mixing together appropriate ingredients that can be formed, introduced in the form of a paste, and that have the ability to bind after implantation in the body for many years—they have the main material that binds the endoprosthesis to the bone. Among polymeric bone cements, poly(methyl methacrylate) (PMMA) is the most widely established material and has a long history of clinical use [9,10,11,12,13,14]. Depending on the intended application, its relatively high stiffness may represent an advantage or a limitation. The Young’s modulus of PMMA is approximately 2–3 GPa, compared with 7–25 GPa for cortical bone and 20–800 MPa for cancellous bone [9,15,16]. Thus, PMMA is substantially stiffer than cancellous bone. Literature reports indicate that the pronounced stiffening produced by PMMA following vertebroplasty may contribute to altered load transfer and an increased risk of adjacent vertebral fractures [9,15,17,18,19,20,21]. Other reported limitations of acrylic bone cements include low fracture toughness, polymerization shrinkage, a pronounced exothermic polymerization reaction, potential toxicity of residual monomer, hypersensitivity to selected components, and limited intrinsic bioactivity [9,17,22,23,24,25,26,27,28]. Alternative polymeric systems with lower stiffness and different curing characteristics have, therefore, been investigated. Polysiloxane-based materials represent one such approach and have been reported to exhibit mechanical characteristics more similar to bone tissue than conventional acrylic cements [20,29,30,31,32,33].

These considerations have also stimulated interest in PU-based biomaterials whose mechanical and physicochemical properties can be tailored over a broad range. Although such systems may potentially be developed for injectable bone applications, their suitability as bone cements requires application-specific evaluation. PUs demonstrate cytocompatibility [21,34], can be functionalized to improve biological interactions [23,25,35], and may exhibit controlled degradation kinetics [26]. PUs owe their mechanical strength primarily to hard segments, whereas soft segments contribute flexibility and elasticity [36]. By controlling the relative content of these segments, materials with a broad range of physicochemical and mechanical properties can be obtained. Due to their physicochemical properties, including strength, elasticity, and wear resistance, as well as their biological characteristics, PUs have been widely investigated for medical applications [37,38,39,40,41,42,43,44]. Currently, PU applications in tissue engineering include the development of scaffolds designed to mimic selected characteristics of natural tissues [45].

To improve the properties of scaffolds, various fillers are used. In the case of PU based on polycaprolactone diol (PCL diol), one of the commonly used fillers is hydroxyapatite (HAp). Asefnejad et al. investigated the effect of the nanofluorohydroxyapatite content on the properties of polyurethane-based materials [46], while H. Liu et al. examined the influence of HAp content on their properties. Other fillers added to the PU matrix include bioglass, beta-calcium triphosphate (ß-TCP), gelatin, cellulose, potassium carbonate, and collagen [46,47,48,49,50,51].

PUs with a porous structure are widely used due to their biocompatibility, well-developed structure, and flexibility, which enable them to be used as bone scaffolds or other bone-replacement materials [52]. They are obtained through a foaming process that creates internal pores, which are crucial for tissue contact and gas exchange. PU foams can be produced using two methods: physical and chemical foaming. In the physical method, the heat generated by the exothermic reaction during PU synthesis is used to vaporize a highly volatile inert liquid (such as fluorotrichloromethane) present in the reaction medium [53]. In the chemical method, water is most commonly added to the reaction medium, where it reacts with the isocyanate groups to generate CO_2_, which acts as a foaming agent. In most cases, water is added directly during the polymerization process. The amount of water added and the conditions under which the process is carried out are critical parameters [54,55]. However, the literature lacks information on phosphate ceramics that could serve a dual role: as a bioactive component and as a carrier of a controlled amount of water acting as a foaming agent. As a result, the obtained material, once introduced into the body, could function as a bone cement with a highly developed microstructure.

In the approach investigated in the present study, microhydroxyapatite (μHAp) was considered not only as a bone-related ceramic component but also as a water-carrying ceramic phase involved in the chemical foaming process. Its highly developed porous structure, high specific surface area, and affinity for water provide the basis for its proposed role as a carrier of water available during PU crosslinking. This water can react with residual isocyanate groups, generating CO_2_ in situ and thereby promoting pore formation. Importantly, μHAp itself is not considered the blowing agent. Rather, it acts as a bioactive ceramic and a water-carrying phase facilitating chemical foaming, whereas the generated CO_2_ acts as the actual blowing agent. The aim of this study was to investigate the effect of PU composition, with particular emphasis on the hard-to-soft segment ratio, on the morphology, chemical structure, physicochemical properties, mechanical performance, and in vitro cytotoxicity of biodegradable PCL diol-based PU intended for bone tissue engineering. PUs with different hard segment contents were compared to identify a formulation suitable for further development as a porous polymer–ceramic composite. In addition, two post-synthesis incubation temperatures (80 °C and 40 °C) were investigated to determine their effect on the resulting material properties. Based on this screening, the selected PU formulation was subsequently combined with μHAp and zirconium oxide (IV) (ZrO_2_) to obtain porous PU/ceramic composites. μHAp was incorporated as a bioactive ceramic and a water-carrying phase promoting in situ CO_2_ generation, whereas ZrO_2_ was introduced with the intention of providing X-ray contrast. However, because both ceramic phases were incorporated simultaneously, a combined contribution of μHAp and ZrO_2_ to the foaming process cannot be excluded, and their individual effects on pore formation cannot be unequivocally distinguished. Their further development toward injectable systems for vertebral augmentation would require additional application-specific characterization, including injectability, rheological behavior, working and setting characteristics, curing exotherm, and radiopacity. These aspects are planned to be investigated in future studies.

## 2. Materials and Methods

### 2.1. Materials

Materials for PU synthesis: Poly(ε-caprolactone) diol (PCL diol), M**_w_** = 2000 g/mol, 1,6-hexamethylene diisocyanate (HDI), M**_w_** = 168.2 g/mol, and 1,4-butanediol (BDO) 99% were obtained from Fisher Scientific (Hampton, VA, USA).

Ceramics for the preparation of the composite:

- Materials for microhydroxyapatite (μHAp) synthesis: calcium hydroxide, orthophosphoric acid, and ammonia 25% were provided by Chempur, Piekary Slaskie, Poland.
- Zirconium (IV) oxide (ZrO_2_) in the form of nanoparticles < 100 nm was obtained from Sigma Aldrich (Saint Louis, MO, USA).

Materials for analysis: Diiodomethane 99% was obtained from Sigma Aldrich (Saint Louis, MO, USA). The antibiotic mixture, penicillin (5000 U)/streptomycin (5 mg/mL)/neomycin (10 mg/mL), was obtained from Sigma Aldrich (Saint Louis, MO, USA). Fetal bovine serum (FBS) was obtained from BioWest (Nuaille, France). McCoy’s 5A culture medium was obtained from VWR (Radnor, PA, USA). Trypsin at 0.25% concentration was obtained from Biological Industrials (Kibbutz Beit Haemek, Israel) and tryptane blue from Gibco (Carlsbad, CA, USA). MTT Cell Viability Assay Kit reagent was obtained from Biotium Inc. (Fremont, CA, USA).

#### 2.1.1. Preparation of Microhydroxyapatite Powders

Microhydroxyapatite (μHAp) powders were obtained by the wet precipitation method developed by Afshar’s group [56]. The synthesis of micro-HAp was performed according to a 5-step procedure described in the work of Laska-Lesniewicz et al. [57]. μHAp with a particle size of 50–75 μm was selected for this work. Previous studies by A. Sobczyk-Guzenda et al. [58] showed that the specific surface area of μHAp was 110.3 m^2^/g. In turn, the volume of pores with diameters from 0.85 to 70 nm is 0.56 cm^3^/g. The obtained μHAp was dried using an ATS120 moisture analyzer (AXIS Sp. z o.o., Gdańsk, Poland) at 160 °C for 120 min, until a constant mass was reached. Based on thermogravimetric analysis (TGA), this drying step resulted in the removal of readily desorbed surface moisture. A further mass loss of approximately 4–4.5 wt% was observed upon heating up to approximately 400 °C. Considering the temperature range of this process and the occurrence of subsequent mass loss at higher temperatures, the mass loss observed up to approximately 400 °C was attributed predominantly to water retained within the porous μHAp structure. This interpretation is also consistent with the high specific surface area and developed pore structure of the obtained μHAp, which favor water retention. Importantly, the water associated with the porous μHAp phase is distinguished here from the structural hydroxyl groups (OH^−^) of HAp, which are not considered equivalent to molecular water.

#### 2.1.2. Preparation of Zirconium Dioxide Powders

Commercial nano-ZrO_2_ was also pre-dried in a vacuum dryer (Goldbrunn, Germany) at 70 °C for 5 h. Before use, it was additionally dried on an AXIS ATS120 moisture analyzer (AXIS Sp. z o.o., Gdańsk, Poland) at 160 °C for 120 min until a constant mass was reached and was subsequently stored in a desiccator. The specific surface area of this ceramic was 50 m^2^/g. This was the time needed to obtain a constant mass. The water content of the dried ZrO_2_, determined by TGA, was below 0.5 wt%.

#### 2.1.3. Synthesis of Polyurethanes

The materials were synthesized using a prepolymer method according to the reaction scheme shown in Figure 1.

Prior to polyurethane synthesis, PCL diol was dehydrated at 120 ± 2 °C for 120 min under vacuum. The first stage of the prepolymer synthesis consisted of adding HDI to liquid PCL diol at 80 ± 2 °C. This step was carried out under flowing nitrogen at 80 ± 2 °C for 180 min. Subsequently, the nitrogen atmosphere was replaced by vacuum, and the prepolymer was intensively stirred under vacuum at 80 ± 2 °C for 60 min. In the second stage, BDO was added as a chain extender at 60 ± 2 °C. The mixture was intensively stirred and then degassed under vacuum for 12 min. For composite samples (PU_3c), after vacuum mixing, ceramic fillers were added and mixed for 5 min. The formulation contained 5 wt% μHAp and 15 wt% ZrO_2_. ZrO_2_ was incorporated with the intention of providing X-ray contrast. Radiopacity was not directly evaluated in the present study. The prepared polymer was poured into silicone molds and incubated at 80 ± 2 °C or 40 ± 2 °C for 24 h. The temperature of 40 °C was selected as a mild processing temperature close to physiological conditions and substantially lower than the conventional post-synthesis incubation temperature of 80 °C, in order to evaluate the ability of the system to crosslink under reduced thermal conditions.

Chemical foaming was based on the reaction between water associated with the ceramic phase and residual isocyanate groups. μHAp was considered the predominant water-carrying ceramic phase because of its developed porous structure, high specific surface area, and substantially higher water-associated fraction compared with ZrO_2_. Water available during crosslinking can react with residual isocyanate groups and generate CO_2_ in situ. Thus, μHAp itself is not regarded as the blowing agent. The generated CO_2_ is the actual blowing gas. The reaction pathway is shown in Figure 2, and the subsequent reaction leading to urea formation is shown in Figure 3 [59].

In the present experimental design, μHAp and ZrO_2_ were incorporated simultaneously into the PU matrix. Separate PU/μHAp and PU/ZrO_2_ control formulations were not investigated. Therefore, although the markedly different water contents and surface characteristics support the proposed predominant contribution of μHAp as the water-carrying ceramic phase, the individual effects of μHAp and ZrO_2_ on the final composite properties cannot be fully separated. The resulting porous composite samples were designated PU_3c. Table 1 shows the synthesis parameters and the molar ratios of the synthesized PUs.

### 2.2. Methods

#### 2.2.1. Surface Morphology Study Using SEM

The surface morphology of the polyurethanes was examined using scanning electron microscopy (SEM) with a JEOL JSM-6610LV scanning electron microscope (JEOL Ltd., Tokyo, Japan). The SEM observations were performed at an accelerating voltage of 10 kV and a chamber pressure of 50 Pa. The beam current (spot size) was adjusted to obtain optimal image sharpness.

#### 2.2.2. Chemical Structure Analysis Using Fourier-Transform Infrared Spectroscopy (FTIR)

The chemical structure of the polyurethanes was analyzed using a Nicolet iS50 FTIR spectrometer (Thermo Fisher Scientific, Waltham, MA, USA) equipped with a Seagull DRIFT reflection/scattering accessory with a variable angle of incidence and a highly sensitive mercury cadmium telluride (MCT) detector. FTIR spectra were recorded in absorbance mode over the wavenumber range of 4000–400 cm^−1^ at a resolution of 4 cm^−1^ and an IR beam incidence angle of 45°. A total of 128 scans were collected for each spectrum. The spectral region between 1800 and 1600 cm^−1^ was deconvoluted using Gaussian peak fitting in OMNIC software, version 9.2 (Thermo Fisher Scientific).

#### 2.2.3. Thermal Characterization by Differential Scanning Calorimetry (DSC) Analysis

Thermal analysis of the tested polyurethane materials was performed using the DSC technique on a DSC 2920 differential scanning calorimeter (TA Instruments, New Castle, DE, USA). The measurement consisted of heating the samples to 250 °C, cooling to −60 °C, and reheating to 250 °C at a rate of 10 °C/min. The measurement was performed in a nitrogen atmosphere. Based on the obtained data, the degree of crystallinity (Xc) of the soft PCL segments was determined using the following formula:

(1) $$X_{c}=\frac{∆H_{m}}{(1-w-u)∆H_{m}^{0}}\cdot 100\%,$$

where: ∆H_m_—heat of fusion of soft segments, w—mass fraction of Hap, u—mass fraction of hard segments, and ∆H^0^_m_—heat of fusion of 100% crystalline PCL, equal to 136 J/g [44,45].

#### 2.2.4. Contact Angle Measurements

Surface wettability was characterized by contact angle measurements using a K100 MKII tensiometer (KRÜSS GmbH, Hamburg, Germany). Water and diiodomethane were selected as the polar and nonpolar probe liquids, respectively, and a droplet volume of 0.8 μL was used for each measurement. The obtained contact angle values were subsequently used to determine the surface free energy (SFE), including its dispersive and polar contributions, according to the Owens–Wendt–Rabel–Kaelble approach. The surface tension components used in the calculations were 21.8 and 51.0 mJ/m^2^ for the dispersive and polar components of water, respectively, and 50.8 and 0 mJ/m^2^ for the corresponding components of diiodomethane [60]. Measurements were performed on three selected samples, with measurements taken at two different locations on each sample.

#### 2.2.5. Hardness Test

The hardness of the manufactured materials was assessed in accordance with the standard PN-ISO 868(Polish Standards—International Organization for Standardization) [61] using a MC-DX/A hardness tester (Max: Control, Racibórz, Poland) on the Shore A scale. The measurements were performed on four selected samples, with five measurements taken at different locations on each sample. The measurements were performed 24 h after sample preparation.

#### 2.2.6. Mechanical Properties Analysis

The mechanical properties of the fabricated composites were evaluated by static compression testing using a Multipurpose Servohydraulic Universal Testing Machine Type LFV-50 kN, Hydraulic Power Pack Type PAC-6, and Digital Material Testing Control System Type PCS5000 (Walter + Bai, Löhningen, Switzerland). Measurements were conducted at a compression speed of 0.1 mm/s and at a temperature of 22 ± 2 °C. Cylindrical specimens with dimensions of 10 × 10 mm were tested. No preload was applied prior to the measurement. The test was repeated six times. Due to the nonlinear mechanical response observed in the tested specimens, Young’s modulus was determined from the initial region of the stress–strain curve. The initial nonlinear portion of the curve, attributed to specimen–platen contact and seating effects resulting from minor surface irregularities, was excluded from the analysis. The subsequent region exhibiting the highest degree of proportionality between stress and strain was identified by inspection of the recorded stress–strain curve, and a linear regression was fitted to this region. The slope of the resulting regression line was taken as the Young’s modulus of the material. The final Young’s modulus was reported as the mean value obtained from six measurements.

#### 2.2.7. Examination of Cell Cytotoxicity

Cytotoxicity was assessed using the MTT assay and the Saos-2 osteoblastic cell line (ATCC^®^ HTB85). Saos-2 cells, derived from a primary osteosarcoma bone tumor, were characterized by G. A. Rodan et al. [62]. Cells were purchased from ATCC (Manassas, VA, USA). The test was performed according to ISO 10993-5 [63]. PU samples were sterilized by radiation with a dose of 25 kGy. An extract (2 mg/mL) was then prepared. Test samples were incubated in McCoy’s culture medium (ATCC) containing no supplements for 48 h at 37 °C. Cells were seeded into a 96-well test plate at a density of 1 × 10^4^ cells per well. Cell culture was carried out using McCoy’s medium containing 1.5 mM L-glutamine and 2200 mg/L sodium bicarbonate, and fetal bovine serum (FBS; Biological Industries) to a final concentration of 15%. Cell culture was carried out under standard conditions (37 °C and a humidified atmosphere of 5% CO_2_ in air). After 24 h of culture, the culture medium was removed and replaced with extracts. The positive control was the cells cultured in McCoy’s medium supplemented with DMEM for the last 24 h, while the negative control was the cells cultured in medium without the addition of any reagents. After 24 h of treatment, the culture medium was removed, and a solution of MTT in culture medium (1 mg/mL, Sigma Aldrich) was added to each well and incubated for 4 h in a CO_2_ incubator under standard conditions. The MTT solution was then replaced with 100 μL of isopropanol (Sigma Aldrich). Absorbance was measured at 570 nm and 650 nm for the reference using a microplate reader Victor X4 (Perkin Elmer, Shelton, CT, USA). Cell viability was determined as a percentage of the negative control.

#### 2.2.8. Molecular Weight Analysis of Selected Samples by Gel Permeation Chromatography

The molecular weight and molecular weight distribution of selected samples were determined by gel permeation chromatography (GPC). GPC analysis was limited to the solid PU samples to directly characterize the molecular weight parameters of the polymer without the additional sample preparation and separation steps required for the ceramic-containing composites. The GPC system consisted of an Agilent Technologies 1260 Infinity pump (Agilent, Santa Clara, CA, USA), an Agilent Technologies 1100 Series autosampler (Agilent), and an Optilab T-rEX refractive index detector (Wyatt Technology, Santa Barbara, CA, USA). HPLC-grade dimethylformamide (DMF) was used as the mobile phase at a flow rate of 0.8 mL/min.

#### 2.2.9. Determination of Curing Time

The curing time of the prepared composites was evaluated in a SUP laboratory oven (Wamed, Warsaw, Poland) maintained at 80 and 40 ± 1 °C. Cylindrical specimens with dimensions of 20 × 5 mm were used for the measurements. The curing time was determined using a needle, which was carefully lowered onto the surface of the specimen without applying additional pressure. This procedure was repeated at 120 s intervals until no visible indentation was observed on the material surface. The measurement was performed in triplicate. Additionally, Shore hardness was measured during the curing process according to the procedure described in Section 2.2.5. The hardness value measured 24 h after sample preparation was used as the reference value. The curing process was considered complete when the measured Shore hardness reached a value within 10% of the hardness determined after 24 h. The measurements were performed in triplicate.

#### 2.2.10. Open Porosity Measurement

The open porosity, P_open_, of the obtained polyurethanes was measured using a method based on Archimedes’ law [64,65]. For this purpose, an analytical balance from Axis was used together with additional equipment. The accuracy of the balance was 0.1 mg, and all measurements were made in deionized water. First, the sample was weighed in air. Then, the sample was placed in a vessel that was connected to a membrane vacuum pump, and the air was removed from the vacuum pores. The air was removed for 15 min under a vacuum of 10^−2^ Pa. Water was then introduced into the vessel through a special valve. In this way, the water filled the open pores in the sample. The next step was to weigh the sample immersed in the water and after removal from the water. Measurements were taken three times for each sample. Equation (2) was used to calculate the porosity:

(2) $$P_{open}=\frac{m_{wet}-m_{a}}{m_{a}-m_{w}},$$

where: m_wet_—mass of the wet sample (soaked in water), measured in air, m_a_—mass of the dry sample, measured in air, and m_w_—mass of the wet sample, measured in water.

The Archimedes-based method provides quantitative information on open porosity. SEM observations were used for qualitative assessment of pore morphology. Quantitative pore-size distribution and pore interconnectivity were not determined in the present study.

#### 2.2.11. Short-Term Stability Assessment

The short-term stability of selected PU materials, namely, the solid PU_3_40 and PU_3_80 samples and the porous composite PU_3c_40 and PU_3c_80 samples, was evaluated under in vitro incubation conditions. The samples were placed individually in polypropylene tubes and immersed in phosphate-buffered saline (PBS). The volume of PBS was adjusted to the mass of each sample using a solution-volume-to-sample-mass ratio of 30 mL/g, according to ISO 13781:2017(E) [66]. The samples were incubated at 37 °C for 30 days in a Heratherm IMC18 incubator (Thermo Fisher Scientific, Hampton, VA, USA). After incubation, material stability was assessed based on mass loss and changes in Young’s modulus. Both mass loss determination and Young’s modulus measurements were performed using six independent specimens for each material. Mass loss was determined gravimetrically using an analytical balance with a readability of 0.1 mg. The initial dry mass of each specimen was recorded before incubation. After 30 days of incubation, the specimens were removed from PBS, gently rinsed with deionized water, and dried under vacuum at 40 °C until a constant mass was reached. The final dry mass was then recorded using the same analytical balance. Mass loss was expressed as the percentage decrease in the dry mass of the specimen relative to its initial dry mass. Additionally, changes in molecular weight parameters, including the number-average molecular weight (Mn), weight-average molecular weight (Mw), and polydispersity index (PDI), were evaluated for the solid PU_3_40 and PU_3_80 samples using gel permeation chromatography (GPC). GPC analysis was performed on one specimen of each material. Young’s modulus and molecular weight parameters were determined according to the experimental procedures described in the corresponding sections above. The obtained values were compared with those determined for the corresponding samples before incubation.

#### 2.2.12. Statistical Analysis

Data obtained from replicate measurements are expressed as mean ± standard deviation (SD). Statistical analysis of the biological data obtained from the MTT assay was performed using Microsoft Excel with the Data Analysis ToolPak. Differences between the tested groups in the MTT assay were evaluated using one-way analysis of variance (ANOVA), followed by the Bonferroni post hoc test for pairwise comparisons. The significance level was set at α = 0.05, and differences with *p* < 0.05 were considered statistically significant.

## 3. Results

### 3.1. Solid Polyurethanes

All PU samples were produced using the prepolymer method. In the present study, the two-step method was selected because one of the potential applications of this material is the development of a two-component system that can be injected into a bone defect site. In addition, an important advantage of this manufacturing process is the reduction of polymerization shrinkage and a significant decrease in the exotherm of the crosslinking process, allowing the process to be carried out at a temperature that is safe for the human body.

#### 3.1.1. Surface Morphology Study Using Scanning Electron Microscopy

Figure 4 shows the SEM micrographs of all non-porous polyurethane samples. SEM analysis was performed on PCL-diol-based PUs with different hard segment contents incubated at 80 °C (samples PU_1_80 to PU_5_80) and at 40 °C (sample PU_3_40) to evaluate the effects of HS content and incubation temperature on the morphology of the obtained materials. The results of the evaluation of the surface morphology of PUs show subtle differences among the obtained SEM images. Samples PU_1_80 and PU_5_80, with the lowest and highest hard segment contents, respectively, exhibited the smoothest and most homogeneous surface microstructures. In contrast, the incubation temperature did not affect the morphology of the examined surfaces.

#### 3.1.2. Chemical Structure Analysis Using FTIR

The FTIR spectra for all samples incubated at 80 °C and for the PU_3_40 sample are shown in Figure 5.

A number of characteristic bands related to the presence of ester bonds, urethane bonds, and aliphatic groups can be distinguished in all FTIR spectra of PUs obtained from PCL diol, HDI, and BDO. Their position, shape, and intensity also change depending on the proportion of hard and soft segments [67]. The bands most characteristic of PU are those associated with the presence of urethane groups (-NH-CO-O-), which are formed by the reaction of isocyanates with PCL diol [68]. Three important bands, the so-called amide bands, are associated with these groups: type I amide (1735–1670 cm^−1^), type II amide (1560–1540 cm^−1^), and type III amide (1270–1280 cm^−1^). The amide type I band is mainly due to stretching vibrations of C=O bonds in the urethane groups. The amide type II band is related to the bending vibrations of N-H group and the stretching vibrations of C-N bonds. Amide band type III, which has a lower intensity, results from a combination of N-H torsional and C-N stretching vibrations [69]. Particularly pronounced changes among the PU_1_80–PU_5_80 samples were observed in the band corresponding to amide I vibrations. The positions of the individual peaks within this broad band are shown in Table 2.

Within this band, a signal corresponding to the C=O stretching vibration of the ester group in PCL diol can also be observed. It occurs in the spectra in the range of 1720–1716 cm^−1^. It is most distinct for the sample with the highest soft segment content, PU_1_80. As the hard segment content increases, its intensity decreases markedly and the signal shifts toward lower wavenumbers, which may be related to the formation of interactions between the C=O groups present in the long chains of the soft segments and the increasing number of N–H groups originating from the hard segments [70]. Two other important signals, associated mainly with the mechanical properties, are located at 1735–1725 cm^−1^ and 1695–1671 cm^−1^. These signals correspond to C=O groups in the urethane bonds of the hard segments that are free (non-hydrogen-bonded) and hydrogen-bonded to N–H groups, respectively [70,71]. Their relative intensities and positions vary depending on the sample. Based on the areas determined by deconvolution of these two characteristic bands, the carbonyl hydrogen bonding index (R) and the degree of phase separation (DPS) were calculated using Equations (3) and (4), respectively:

(3) $$R=\frac{C_{bonded}\cdot f_{bonded}}{C_{free}\cdot f_{free}}=\frac{A_{1695-1671}}{A_{1735-1725}},$$

(4) $$PS=\frac{C_{bonded}}{C_{bonded}+C_{free}}-\frac{R}{1+R},$$

where A is the intensity of the characteristic absorbance, C is the concentration, and f bonded and f free are the extinction coefficients of the bonded and the free carbonyl groups, respectively. The value of bonded/free was between 1.0 and 1.2 [69,72]. In this study, the value of bonded/free is taken as 1.0. The R and DPS values are presented in Table 3.

For the samples incubated at 80 °C, the position of the peak corresponding to C=O groups involved in hydrogen bonding with the –NH groups of the hard segments shifted from 1695 cm^−1^ for sample PU_1_80 to 1684 cm^−1^ for sample PU_3_80. This shift indicates an increase in the number of hydrogen bonds, which is further supported by the substantial increase in the R parameter from 0.50 to 2.88. With a further increase in the molar ratio of HDI and the amount of chain extender (1,4-butanediol) relative to the PCL diol content, the position of this peak remained essentially unchanged for the PU_4_80 sample, accompanied by a slight decrease in both the R parameter and the degree of phase separation (DPS). In contrast, for the sample with the highest hard segment content of 32% (PU_5_80), the values of the R and DPS parameters increased again, reaching 3.1 and 0.76, respectively. Moreover, a decrease in the PCL-diol-to-HDI/BDO ratio resulted in a marked reduction in the intensity of this peak and the appearance of additional bands at 1680 and 1671 cm^−1^ for samples PU_4_80 and PU_5_80, respectively. This phenomenon is most likely associated with the formation of new structural arrangements of hard segments linked through hydrogen bonding. In these samples, the shorter polymer chains and the higher hard segment content promoted the formation of stronger hydrogen bonds between the C=O and –NH groups [70]. The areas of these additional bands were also included in the calculation of the R parameter.

Another characteristic absorption band attributed to urethane groups corresponds to the N–H stretching vibration in the 3500–3200 cm^−1^ region. This band changes in width, intensity, and shape depending on the hard segment content. This is because the N–H groups are involved in the formation of hydrogen bonds with both C=O and C–O–C groups. If the N–H group is not involved in hydrogen bonding, the corresponding maximum occurs above 3350 cm^−1^. For most samples this band consists of two signals [73]. Only for sample PU_1_80 is one of these peaks located within the wavenumber range corresponding to non-hydrogen-bonded N–H groups. For the remaining samples, the most intense peak is located at 3326 cm^−1^, which is the typical position for the NH····· O=C group. If this band shifts toward a lower frequency, it may indicate the formation of hydrogen bonds with C–O–C groups [67], as is most likely the case for samples PU_4_80 and PU_5 _80.

The spectra shown in Figure 5 exhibit C–O–C stretching vibration bands in the 1100–1300 cm^−1^ range, characteristic of ester and urethane bonds. The C–O–C bond present in urethane groups may exhibit one or two maxima in the 1000–1100 cm^−1^ range. The presence of two maxima may indicate that the oxygen atom of this group participates in hydrogen bonding with N–H groups [67]. Such a spectral pattern was observed in this region for the samples with the highest hard segment contents, PU_4_80 and PU_5_80.

The chemical structure of the investigated PU contains aliphatic chains derived from PCL, HDI, and BDO. These are reflected in the spectra by two bands corresponding to CH_2_ stretching vibrations in the 2930–2830 cm^−1^ range and CH_2_ deformation bands at approximately 1470 and 1350 cm^−1^ [69,70,71,72,73].

The spectrum of sample PU_3_40, which was incubated at 40 °C, was similar to that of sample PU_3_80 in terms of the position of the characteristic peaks. Only that corresponding to bonds within the soft segment was slightly shifted toward lower wavenumbers. Differences were observed in the relative intensities of the individual bonds originating from both hard and soft segments, which were associated with a decrease in the number of hydrogen bonds and a decrease in the degree of phase separation. The spectrum of this sample is shown in Figure 5c.

All the obtained spectra lacked the band at 2270–2260 cm^−1^ corresponding to the N=C=O group, which may be present in the case of incomplete conversion of HDI isocyanate groups during synthesis [74]. Its absence indicates that the isocyanate groups reacted completely during the polyaddition reaction. Furthermore, in the spectra shown in Figure 3, no bands attributable to the hydroxyl groups of BDO and PCL were observed in the 3200–3600 cm^−1^ range, providing further evidence of the complete reaction of these groups with the isocyanate groups [75].

#### 3.1.3. Thermal Characterization by Differential Scanning Calorimetry (DSC) Analysis

The thermal properties of the produced PCL-diol-based PUs were assessed using DSC. The characteristic melting temperatures (T_m_) and melting enthalpies of the soft and hard segments were determined from the obtained thermograms. X_c_ of the soft segments was also calculated, as shown in Table 4.

Based on the obtained results, it can be concluded that, as the hard segment content in PCL-diol-based polyurethanes incubated at 80 °C increased, the T_m_ and ΔH_m_ corresponding to the soft segments decreased, whereas the T_m_ and ΔH_m_ corresponding to the hard segments increased. The ΔH_m_ of the soft segments was used to calculate their X_c_, as shown in Table 4. Due to equipment limitations, the T_g_ values being below −50 °C could not be determined. The results obtained for samples incubated at 80 °C indicate that an increase in the hard segment content was accompanied by a decrease in the X_c_ of the soft segments, which in turn led to a decrease in the T_m_ from 42.6 to 26.5 °C, i.e., to a temperature close to the melting temperature of PCL, which is consistent with literature reports [76]. In contrast, for all samples, ΔH_m_ of the hard segments increased from 16.0 J/g for PU_1_80 to 27.4 J/g for PU_5_80, while the T_m_ increased from 121.7 to 147.0 °C.

In the case of the PU_3_40 polyurethane sample, the melting temperatures and ΔH_m_ of both the hard and soft segments were lower than those of the PU_3_80 sample, which had the same hard segment content but was incubated at 80 °C. This observation is consistent with the results obtained from the FTIR analysis of the chemical structure.

#### 3.1.4. Surface Free Energy Determined Based on Surface Wettability Measurement

The values of surface free energy and its components determined based on the measurement of water and diiodomethane contact angles are shown in Figure 6.

The PCL-diol-based polyurethanes exhibited hydrophilic surface characteristics, as their water contact angles were below 90°. All the produced PCL-diol-based polyurethanes exhibited surface free energy (SFE) values above 30 mJ/m^2^. Changes in the molar ratios of PCL diol, isocyanate, and chain extender resulted in changes in the polar and dispersive components and, consequently, in the total SFE values. The polar component of the SFE for the samples with the highest hard segment content was 2.3 and 2.9 mJ/m^2^, whereas the highest value of this component, 12 mJ/m^2^, was recorded for PU_2_80. The post-synthesis incubation temperature did not affect either the SFE or the water contact angle.

#### 3.1.5. Hardness

The hardness of the produced PCL-diol-based polyurethane samples was assessed using the Shore A method, and the results are shown in Figure 7. The polyurethane with the lowest hard segment content exhibited the lowest hardness, with a value of 87.7 ± 1.5 °ShA. A change in the molar ratio, and consequently an increase in the hard segment content, resulted in an increase in polymer hardness. The highest hardness, 98.0 ± 1.3 °ShA, was recorded for PU_5_80, which had a hard segment content of 32%. The post-synthesis incubation temperature did not affect the hardness of the resulting polymer.

#### 3.1.6. Static Compression Test

The Young’s modulus values determined by static compression testing are shown in Figure 8. The lowest value of Young’s modulus, 27.2 ± 3.8 MPa, was recorded for PU_1_80. An increase in the hard segments of the PU was accompanied by an increase in Young’s modulus. The post-synthesis incubation temperature had no effect on the mechanical properties. Increasing the hard segment content resulted in a progressive increase in Young’s modulus. The highest values were obtained for PU_3_80 and PU_5_80, reaching 36.9 ± 3.5 MPa and 41.0 ± 2.9 MPa, respectively.

#### 3.1.7. Biological Tests

The cytotoxicity of the PCL-diol-based polyurethanes was assessed using the MTT assay. The results presented in Figure 9 showed that, for each PU formulation, the viability of Saos-2 cells was above the 70% threshold specified in ISO 10993-5 [63] for cytotoxicity assessment. Statistical analysis (one-way ANOVA with Bonferroni post hoc test, α = 0.05%) revealed no statistically significant differences in cell viability between the tested PU samples and the negative control. Consequently, all evaluated polyurethane formulations do not exhibit any cytotoxic potential and meet the ISO standard requirements for in vitro cytocompatibility.

#### 3.1.8. Molecular Weight and Molecular Weight Distribution by GPC

Gel permeation chromatography (GPC) analysis showed that both PU_3 samples had typical molecular weights for similar syntheses [77]. The molecular weight parameters determined by GPC analysis are summarized in Table 5. For sample PU_3_40, the number-average molecular weight (M_n_) and weight-average molecular weight (M_w_) were 44,300 and 80,620 g/mol, respectively, and the polydispersity index (PDI) was 1.819. Increasing the final incubation temperature from 40 to 80 °C resulted in an increase in M_n_ to 49,500 g/mol and a slight increase in M_w_ to 82,170 g/mol. At the same time, PDI decreased to 1.660. Thus, the higher incubation temperature caused only a relatively small change in M_w_ (approximately 2%), while the increase in M_n_ was more pronounced (approximately 12%). The simultaneous increase in M_n_ content and decrease in PDI content indicates a narrower molecular weight distribution in PU_3_80 and a reduced share of the low-molecular-weight polymer fraction.

#### 3.1.9. Assessment of Curing Time

The curing time was determined for the selected PU_3_40 and PU_3_80 samples. The curing time of PU_3_40 was 40 ± 5 min, which was 20 ± 5 min shorter than that of PU_3_80. The curing time was defined as the time required for each sample to reach a Shore A hardness of 80°.

### 3.2. Porous PU with the Addition of Ceramics

For the synthesis of porous PU, samples of PU_3 composition were selected and incubated at 40 and 80 °C. This formulation exhibited one of the highest degrees of phase separation among the investigated materials and favorable mechanical properties, while retaining sufficient flexibility to enable incorporation of the ceramic phase. Porous composites were prepared by simultaneously incorporating μHAp and ZrO_2_. The proposed foaming mechanism involves water associated with the ceramic phase reacting with residual isocyanate groups and generating CO_2_ in situ, with the generated CO_2_ acting as the actual blowing gas. Based on its developed porous structure, high specific surface area, and higher water-associated fraction compared with ZrO_2_, μHAp was considered the predominant water-carrying ceramic phase. However, because μHAp and ZrO_2_ were incorporated simultaneously and separate PU/μHAp and PU/ZrO_2_ control formulations were not investigated, the individual contribution of μHAp to the foaming process cannot be directly demonstrated from the present experimental design. The ceramic phases differed in their physicochemical characteristics. The specific surface area of μHAp was 110.3 m^2^/g, and the pore volume in the pore-diameter range of 0.85–70 nm was 0.56 cm^3^/g. In comparison, ZrO_2_ exhibited a lower specific surface area of 50 m^2^/g. Thermogravimetric analysis showed a water-associated mass loss of approximately 4–4.5 wt% for μHAp upon heating up to approximately 400 °C, whereas the water content of dried ZrO_2_ was below 0.5 wt%. These differences support the proposed predominant contribution of μHAp as the water-carrying ceramic phase.

#### 3.2.1. Surface Morphology

SEM micrographs of the obtained foamed composites containing a total of 20 wt% fillers, at ×500 magnification, are presented in Figure 10.

The incorporation of the μHAp/ZrO_2_ ceramic phase resulted in the formation of a porous PU structure. SEM observations showed qualitatively similar porous morphologies for PU_3c_40 and PU_3c_80, although slightly larger pores were observed for PU_3c_80. This difference may be associated with temperature-dependent changes in reaction kinetics and viscosity during PU crosslinking. However, quantitative pore-size distribution was not determined in the present study. At higher magnification (approximately 1000×), cauliflower-like structures corresponding to aggregates of the ceramic phase were observed. In both composites, the microstructure was heterogeneous and contained these characteristic ceramic-rich features.

#### 3.2.2. Porosity Analysis of the Obtained Materials

Open porosity analysis was determined using the Archimedes’ principle method. The total open porosity of the PU_3c_40 sample was 70.4 ± 1.7%, whereas that of PU_3c_80 was 73.3 ± 1.2%. Both composites contained the same total amount of fillers (20 wt%) and differed in the post-synthesis incubation temperature. The slightly higher open porosity observed for the sample incubated at 80 °C may be associated with temperature-dependent differences in the foaming and crosslinking processes [78].

#### 3.2.3. Chemical Structure Analysis

Figure 11 shows the FTIR spectra obtained for the foamed samples incubated at two different temperatures, PU_3c_40 and PU_3c_80, and for their solid counterparts. Analysis of the spectra revealed that the spectral profiles of the two samples were similar regardless of the incubation temperature. Table 6 presents the absorption bands corresponding to amide I and ester bonds. Although the solid counterpart of PU_3c_80 exhibited a structure containing numerous hydrogen bonds, the structure of PU_3c_80 became similar to the less ordered structure of PU_3c_40 after foaming. Table 7 shows the hydrogen bond indices and the degree of phase separation. The results indicate that the DPS index of the sample incubated at 80 °C decreased from 0.74 to 0.61 after foaming, whereas the sample incubated at 40 °C exhibited a comparable DPS value before and after foaming. This indicates that the chemical structure of sample PU_3_40 is characterized by a comparable number of hydrogen bonds formed between the –NH and C=O groups within the hard segments. In addition, following the introduction of water together with the ceramic phase, bands corresponding to the stretching vibrations of urea groups appeared at 1665 cm^−1^ in the spectra of both porous samples. These bands are typical of disordered hydrogen-bonded urea C=O groups. Peaks corresponding to ordered hydrogen-bonded urea C=O groups were not detected. Among the bands originating from the introduced ceramic phase, the only identified peak was located at 500 cm^−1^ and corresponded to the stretching vibrations of Zr–O bonds [79]. The pronounced intensity of this peak may indicate the formation of ZrO_2_ agglomerates in the PU matrix. In addition, a broad band appeared in the 3400–3500 cm^−1^ region, corresponding to O–H stretching vibrations. These vibrations may originate from hydroxyl groups of the phosphate ceramic as well as from hydroxyl groups attached to the PU chains during the foaming process. Bands corresponding to PO_4_^−3^ groups of HAp [80] were not observed in the spectrum. This may be attributed, firstly, to the low HAp content of 5 wt% and, secondly, to its uniform distribution within the PU matrix.

#### 3.2.4. Thermal Characterization by DSC Analysis

The thermal properties of the porous samples were analyzed by DSC, and the obtained parameters were compared with those of their solid counterparts. The results are summarized in Table 8. The ΔH_m_ of the soft segments in the porous samples was comparable to that of the corresponding solid samples. For the hard segments, the T_m_ of PU_3_40 increased by 3.5 °C relative to PU_3_40, while the ΔH_m_ of this phase remained at a comparable level. These results are consistent with the FTIR analysis, indicating that the hydrogen bonding interactions within the hard segments were largely preserved after foaming. In contrast, for the porous PU_3c_80 sample, both the T_m_ and ΔH_m_ of the hard segments were lower than those of the corresponding non-porous PU_3_80 sample. This indicates a reduction in the degree of organization of the hard segments, which may result from the longer time the material remained in the plastic state during foaming.

#### 3.2.5. Surface Free Energy Determined Based on Surface Wettability Measurement, Static Compression Test, and Biological Tests

A summary of the surface wettability measurements, surface free energy, Young’s modulus in static compression, and extract-based cytotoxicity results using Saos-2 cells is presented in Table 9. Compared with the corresponding solid polyurethane samples, the porous composites exhibited a higher polar component of the surface free energy. The total SFE was 62.0 ± 2.6 mJ/m^2^ for PU_3c_40 and 55.5 ± 3.7 mJ/m^2^ for PU_3c_80. The Young’s modulus of PU_3c_40 was 29.5 ± 2.5 MPa, compared with 31.7 ± 2.7 MPa for PU_3_40, whereas the Young’s modulus of PU_3c_80 was 21.2 ± 3.0 MPa, compared with 36.9 ± 3.5 MPa for PU_3_80. In the extract-based MTT assay, cell viability was 95.6 ± 6.8% for PU_3c_40 and 86.5 ± 7.6% for PU_3c_80. Both values remained above the 70% viability threshold used for cytotoxicity assessment according to ISO 10993-5 [63], indicating that neither porous composite exhibited a cytotoxic effect under the tested extract-based conditions. One-way ANOVA followed by the Bonferroni post hoc test revealed no statistically significant differences in cell viability among the tested samples (*p* > 0.05).

#### 3.2.6. Short-Term Stability of Polyurethane Materials During Incubation

The stability of the selected polyurethane materials was evaluated after 30 days of incubation at 37 °C based on mass loss and changes in Young’s modulus. Additionally, molecular weight parameters (M_n_, M_w_, and PDI) were determined for the solid PU_3_40 and PU_3_80 samples before and after incubation (Table 10).

After 30 days of incubation, all investigated materials exhibited low mass loss, ranging from 0.53 to 0.63%. Only minor changes in Young’s modulus were observed, with the values remaining comparable to those determined before incubation for both the solid and porous composite samples. GPC analysis of the solid PU_3 formulations also revealed only small changes in molecular weight parameters. A slight decrease in M_n_ and M_w_ was observed for both PU_3_40 and PU_3_80, whereas the PDI values remained nearly unchanged.

## 4. Discussion

The main objective of this study was to select the optimum composition of a PU with an appropriate hard-to-soft segment ratio for further development as a solid or porous material for bone tissue engineering, particularly as a potential substitute for cancellous bone. PUs with different hard segment contents were compared in terms of their structural, physicochemical, mechanical, and extract-based cytotoxicity characteristics. Based on this screening, the PU_3 formulation was selected for preparation of porous PU/ceramic composites containing μHAp and ZrO_2_. The ceramic phases were incorporated simultaneously. Therefore, their individual contributions to the final composite properties cannot be unequivocally separated. ZrO_2_ was introduced with the intention of providing X-ray contrast, but radiopacity was not experimentally evaluated in the present study.

The surface morphology of the solid PCL-diol-based PU varied only moderately with hard segment content. FTIR analysis indicated conversion of the reactive isocyanate groups under the applied synthesis conditions, which is relevant because residual isocyanates are undesirable in materials intended for biomedical applications. The polyaddition process was conducted without the use of a catalyst. This is an extremely important feature due to the fact that isocyanates are extremely reactive substances and react very easily with water and protein groups in the epithelium in the blood, causing various allergic reactions, which in consequence can lead to tissue necrosis [81]. Another potential hazard associated with the use of PUs for biomedical applications is the presence of catalysts. In this context, tin-based catalysts and low-molecular-weight amine compounds, which are commonly used in industrial PU synthesis, are not suitable for biomedical applications. Tin, even at low concentrations, may pose a risk to the human body due to its potential to accumulate in various organs [82]. Importantly, curing was also achieved under the reduced-temperature condition of 40 °C. The curing time determined for PU_3_40 was 40 min using the experimental procedure described in Section 2.2.9. This finding demonstrates that curing can proceed at a substantially lower temperature than the conventional 80 °C post-synthesis incubation condition. However, curing at 40 °C alone does not establish suitability as an injectable bone cement, because injectability, rheological behavior, working and setting characteristics, curing exotherm, radiopacity, and performance under simulated application conditions were not evaluated in the present study. These parameters require further application-specific investigation. Moreover, this curing time is comparable to that of the commercially available polysiloxane-based elastomeric bone cement VK-100. These data are based on our own results obtained during comparative tests, in which PUs manufactured in the same way were analyzed. In this study, the HDI:PCL ratios were selected to obtain a hard segment content ranging from 17.6 to 32.0%. One of the objectives of this study was to obtain a Young’s modulus of approximately 30 MPa. This target value was based on our previous studies. An elastic modulus of approximately 30 MPa is comparable to that of cancellous bone, which may potentially reduce the risk of fractures in adjacent vertebral bodies [83,84]. The monomer ratios used resulted in the formation of different numbers of hydrogen bonds within the PU structure. These hydrogen bonds were formed between the –NH groups (proton donors) and the C=O groups (acceptors), both within the same molecule and between individual polymer chains. Hydrogen bonds in the PU structure play a key role in determining its physical, mechanical, and thermal properties [85]. Based on the studies presented in this work, that hydrogen bonds were present not only within the hard segments but also involved groups originating from the soft segments. Based on the deconvolution of the bands corresponding to hydrogen-bonded and free C=O groups, the hydrogen bonding index and the degree of phase separation, which is directly related to the presence of hydrogen bonds, were determined. The degree of phase separation reached one of its highest values (0.74) for the PU_3_80 sample, containing 25.5% hard segments. With a further increase in the hard segment content, this value remained relatively stable and reached its maximum value of 0.76 for the PU_5_80 sample. In addition to the effect of hard segment content, the incubation temperature influenced the structural organization of the PUs. FTIR analysis revealed a slightly higher carbonyl hydrogen bonding index (R = 2.88) and degree of phase separation (DPS = 0.74) for PU_3_80 compared with PU_3_40 (R = 2.62 and DPS = 0.72, respectively). These differences were consistent with the DSC results, which showed higher melting temperatures and enthalpies of both the soft and hard segments for PU_3_80. In particular, the T_m_ of the hard segments increased from 122.7 °C for PU_3_40 to 130.4 °C for PU_3_80, while the corresponding ΔH_m_ increased from 19.3 to 22.2 J/g. Taken together, the GPC, FTIR, and DSC results indicate that incubation at 80 °C promotes a higher degree of structural organization of the PU, reflected in a slightly higher molecular weight, a narrower molecular weight distribution, stronger intermolecular interactions, and slightly greater phase separation. Thus, incubation temperature appears to influence structural organization, intersegment interactions, and molecular weight distribution more strongly than the M_w_ value itself. There are very high values and are characteristic of PUs intended for biomedical applications requiring high mechanical strength [59,85,86,87,88]. For the PU_3_80 sample incubated at 80 °C, one of the two highest Young’s modulus values was obtained, reaching 36.9 MPa. Subsequently, this value remained relatively stable. However, for the PU_5_80 sample, a further increase in the hard segment content resulted in an increase in Young’s modulus to 41 MPa. This increase was accompanied by a significant increase in material hardness. Owing to this phase-separated structure and the high content of hard segments, the material may become brittle under operating conditions, resulting in a reduced capacity for energy absorption and cushioning. Moreover, after foaming, the material may exhibit non-uniform porosity, which could result from the uneven distribution and flow of the blowing gas (CO_2_). Considering the combined structural and mechanical results, PU_3 was selected for further development as the porous composite formulation.

Surface wettability and surface free energy are important physicochemical characteristics of biomaterials because they influence interactions occurring at the material–biological environment interface [89]. In particular, the balance between the polar and dispersive components of surface free energy can affect the interaction of the material surface with water and biological molecules. Studies have indicated that surface wettability and surface free energy may influence protein adsorption and, consequently, subsequent cell–material interactions [90]. Therefore, changes in these surface characteristics are relevant when considering polymeric materials for bone tissue engineering applications. The investigated PCL-diol-based PU showed moderate wettability, while changes in hard segment content were accompanied by changes in the polar and dispersive components of surface free energy. These differences can be related to changes in the chemical composition and organization of the PU surface, including the relative contribution of polar functional groups. For the PU_3 formulation, the post-synthesis incubation temperature produced only limited changes in the measured surface-energy parameters, suggesting that the surface characteristics of this formulation were not strongly affected by the applied incubation temperature. In the porous composites, the incorporation of the μHAp/ZrO_2_ ceramic phase was associated with an increase in the polar component of surface free energy. The presence of ceramic phases containing polar surface groups may contribute to enhanced interactions with water and other polar molecules. From the perspective of biomaterial–tissue interactions, such changes may be considered potentially favorable, since surface polarity and wettability have been reported to influence protein adsorption and subsequent cell–material interactions [90]. However, in the present study, protein adsorption, cell adhesion, proliferation, mineralization, and osteogenic activity were not directly evaluated. Therefore, the observed changes in surface free energy should be interpreted as physicochemical characteristics of the materials rather than as direct evidence of improved biological performance. Dedicated biological studies would be required to establish whether these surface properties translate into enhanced cellular responses.

The biological assessment in the present study was limited to an extract-based MTT cytotoxicity assay using Saos-2 cells. Cell viability remained above the 70% threshold specified in ISO 10993-5 [63] for cytotoxicity assessment for all investigated formulations, indicating the absence of cytotoxic effects under the tested extract-based conditions. This is consistent with literature reports, because polyurethanes are considered to be non-toxic and cytocompatible [91].

Based on the combined structural and mechanical results, PU_3_80 and PU_3_40 were selected for preparation of porous composites containing μHAp and ZrO_2_. The obtained composites exhibited high open porosity (70.4–73.3%), supporting their further consideration as porous materials for bone tissue engineering. The proposed foaming mechanism is based on the reaction of water associated with the ceramic phase with residual isocyanate groups, resulting in in situ CO_2_ generation. The generated CO_2_ is regarded as the actual blowing gas, whereas μHAp is considered a bioactive ceramic and a proposed predominant water-carrying phase because of its developed porous structure, high specific surface area, and higher water-associated fraction than ZrO_2_. The experimentally determined porosity is consistent with effective CO_2_-mediated foaming. The μHAp and ZrO_2_ ceramics were incorporated simultaneously, and changes in the mechanical, surface, and morphological properties of the composites should be interpreted as effects of the combined ceramic phase rather than attributed unequivocally to μHAp alone.

For the composite processed at 40 °C, the degree of phase separation remained at 0.72 relative to the corresponding solid sample, and the Young’s modulus remained comparable to that of the solid PU_3_40 formulation. These observations indicate that formation of the porous composite at the lower processing temperature did not markedly alter these measured structural and mechanical parameters. By contrast, PU_3c_80 exhibited a lower Young’s modulus and a lower DPS than its solid counterpart.

The results obtained after 30 days of incubation indicate good short-term stability of the investigated PU materials under the applied conditions. The low mass loss, not exceeding 0.63%, was accompanied by preservation of the mechanical properties, as indicated by the comparable Young’s modulus values before and after incubation. This suggests that the materials maintained their mechanical integrity during the investigated period. The GPC results for the solid PU_3 formulations were consistent with these observations. Only minor decreases in M_n_ and M_w_ were detected after incubation, while PDI remained essentially unchanged, indicating limited changes in the molecular weight characteristics of the PU during the 30-day period. Overall, the combination of low mass loss, preservation of Young’s modulus, and limited changes in molecular weight parameters supports the short-term stability of the investigated materials at 37 °C. However, these findings should be considered as an assessment of short-term stability rather than a complete characterization of their long-term degradation behavior.

The incorporation of the combined μHAp/ZrO_2_ ceramic phase was associated with an increase in the polar component and, consequently, in the surface free energy of the porous composites. The presence of polar surface groups may favor interactions of the material surface with water and other polar molecules. The extract-based MTT assay demonstrated that the investigated porous composites did not exhibit cytotoxic effects to-ward Saos-2 cells under the tested conditions. Together with their high open porosity and short-term stability, these findings support further investigation of the composites as porous materials for bone tissue engineering, while additional biological studies are required to establish their broader biological performance.

## 5. Conclusions

The present study demonstrated that hard segment content influences the structural, physicochemical, and mechanical properties of PCL-diol-based PU. Among the investigated formulations, PU_3 containing 25.5% hard segments exhibited a favorable combination of structural and mechanical properties and was selected for the preparation of porous polymer–ceramic composites.

The incorporation of μHAp and ZrO_2_ enabled the preparation of highly porous composites with open porosity of 70.4–73.3%. The obtained results are consistent with the proposed role of μHAp as a bioactive ceramic and a predominant water-carrying phase promoting in situ CO_2_ generation during PU crosslinking. The observed effects reflect the contribution of the combined μHAp/ZrO_2_ ceramic phase, while the specific contributions of the individual ceramic components warrant further investigation.

The extract-based MTT assay indicated the absence of cytotoxic effects under the tested conditions.

In addition, the low mass loss and limited changes in mechanical and molecular weight parameters after 30 days at 37 °C supported the short-term stability of the investigated materials.

Overall, the results support the further development of porous PU/μHAp/ZrO_2_ composites as materials for bone tissue engineering, particularly as potential porous substitutes for cancellous bone. Although curing at 40 °C was demonstrated, further development toward injectable systems for vertebral augmentation requires additional application-specific characterization, including injectability, rheological behavior, working and setting characteristics, curing exotherm, radiopacity, and performance under simulated application conditions.

## Figures and Tables

**Figure 1:**
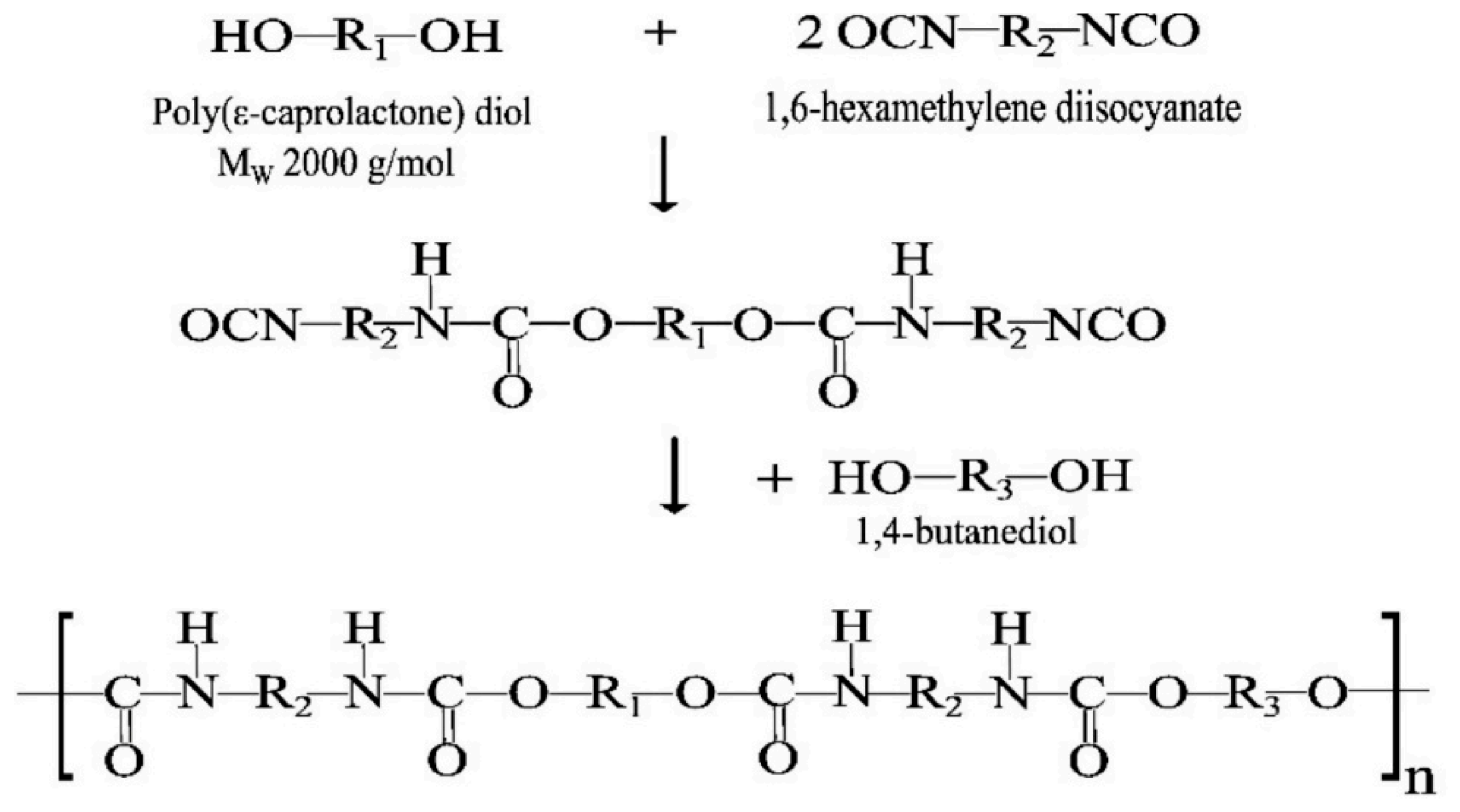
Scheme of the two-step (prepolymer) reaction of polyurethane synthesis.

**Figure 2:**
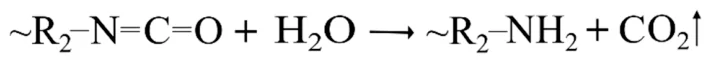
Reaction of the isocyanate group with water.

**Figure 3:**
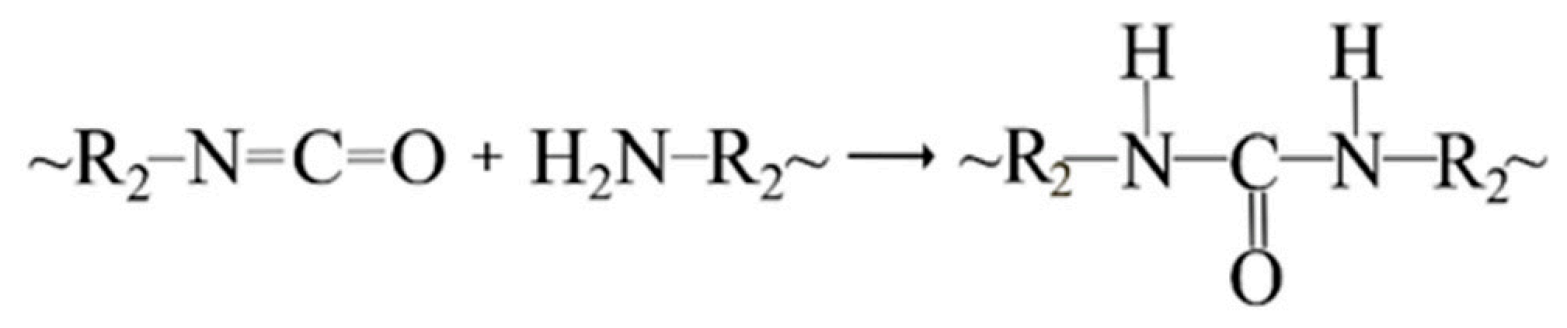
Reaction schemes during crosslinking.

**Figure 4:**
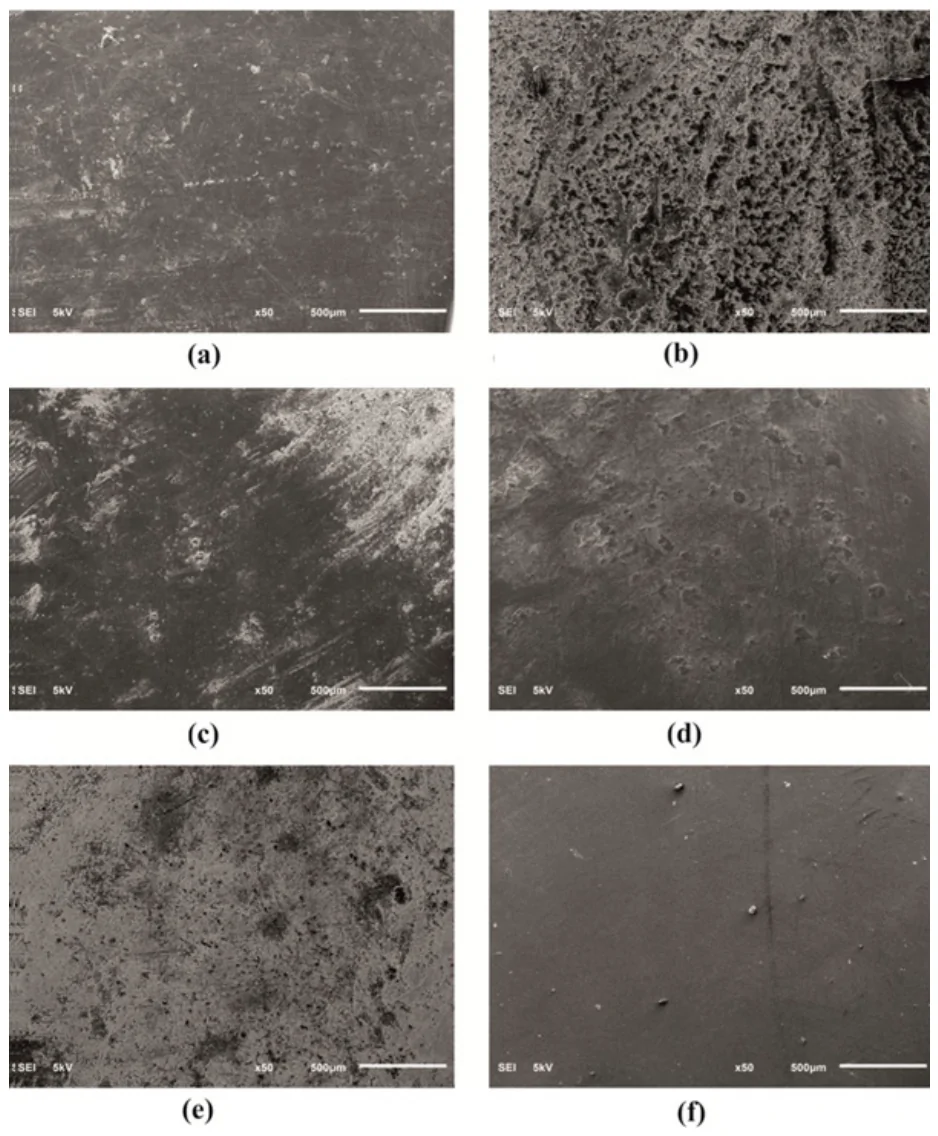
SEM micrographs of PCL-diol-based polyurethanes at ×50 magnification: PU_1_80 (**a**), PU_2_80 (**b**), PU_3_80 (**c**), PU_3_40 (**d**), PU_4_80 (**e**), and PU_5_80 (**f**).

**Figure 5:**
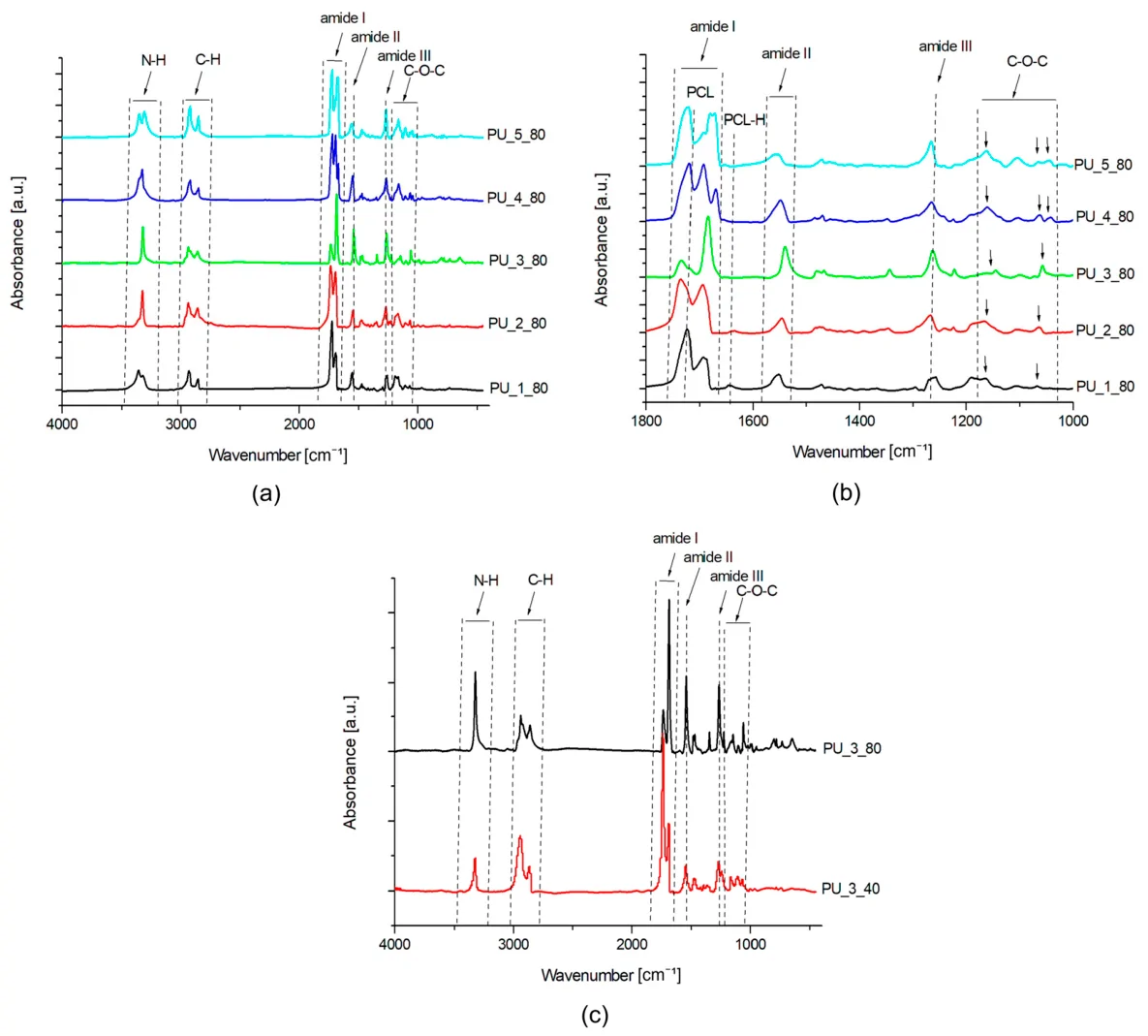
FTIR spectra of samples PU_1_80, PU_2_80, PU_3_80, PU_4_80, and PU_5_80 in the full spectral range of 4000–400 cm^−1^ (**a**), the same samples in a narrowed range of 1800–1000 cm^−1^ (**b**), and samples PU_3_80 and PU_3_40 in the full mid-IR range (**c**).

**Figure 6:**
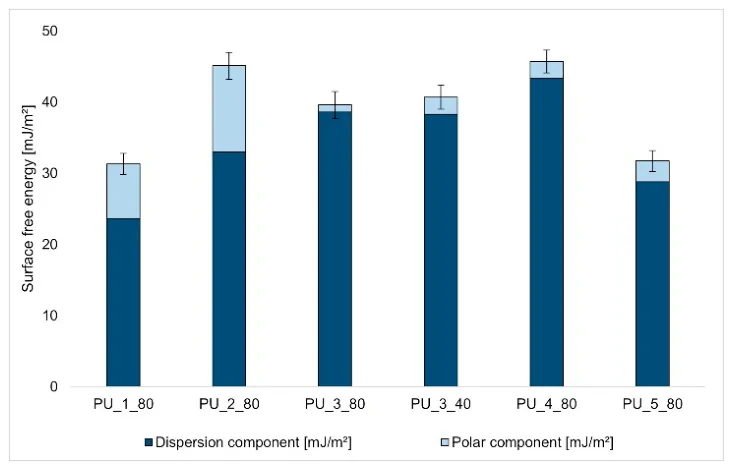
Dispersion component, polarity, and surface free energy for the produced polyurethanes.

**Figure 7:**
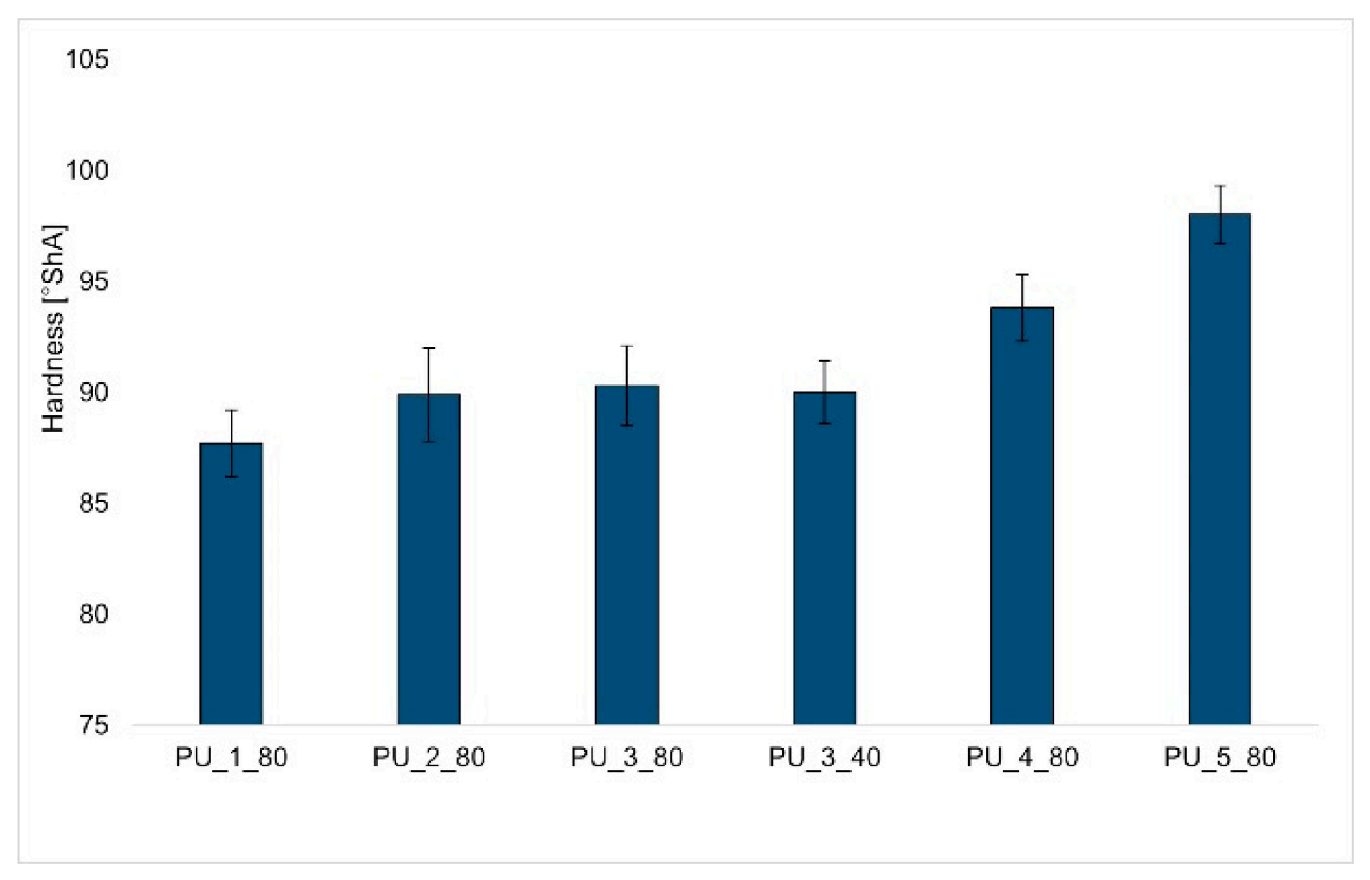
Shore A hardness of the synthesized polyurethanes.

**Figure 8:**
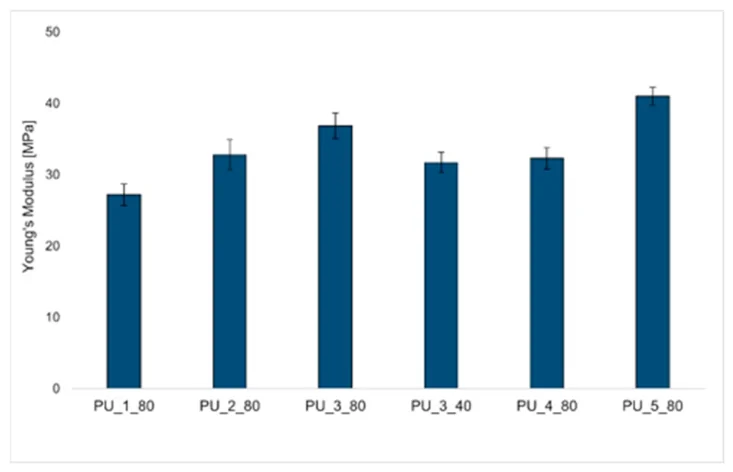
Young’s modulus for PCL-diol-based polyurethanes.

**Figure 9:**
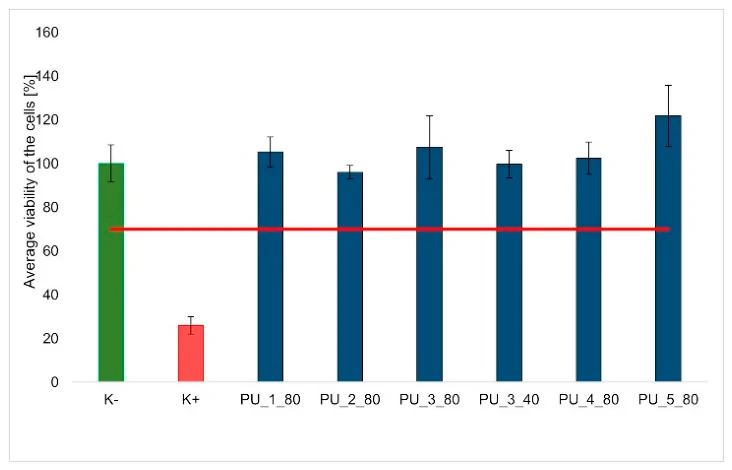
Results of the Saos-2 cell line survival assessment for PCL-diol-based polyurethanes.

**Figure 10:**
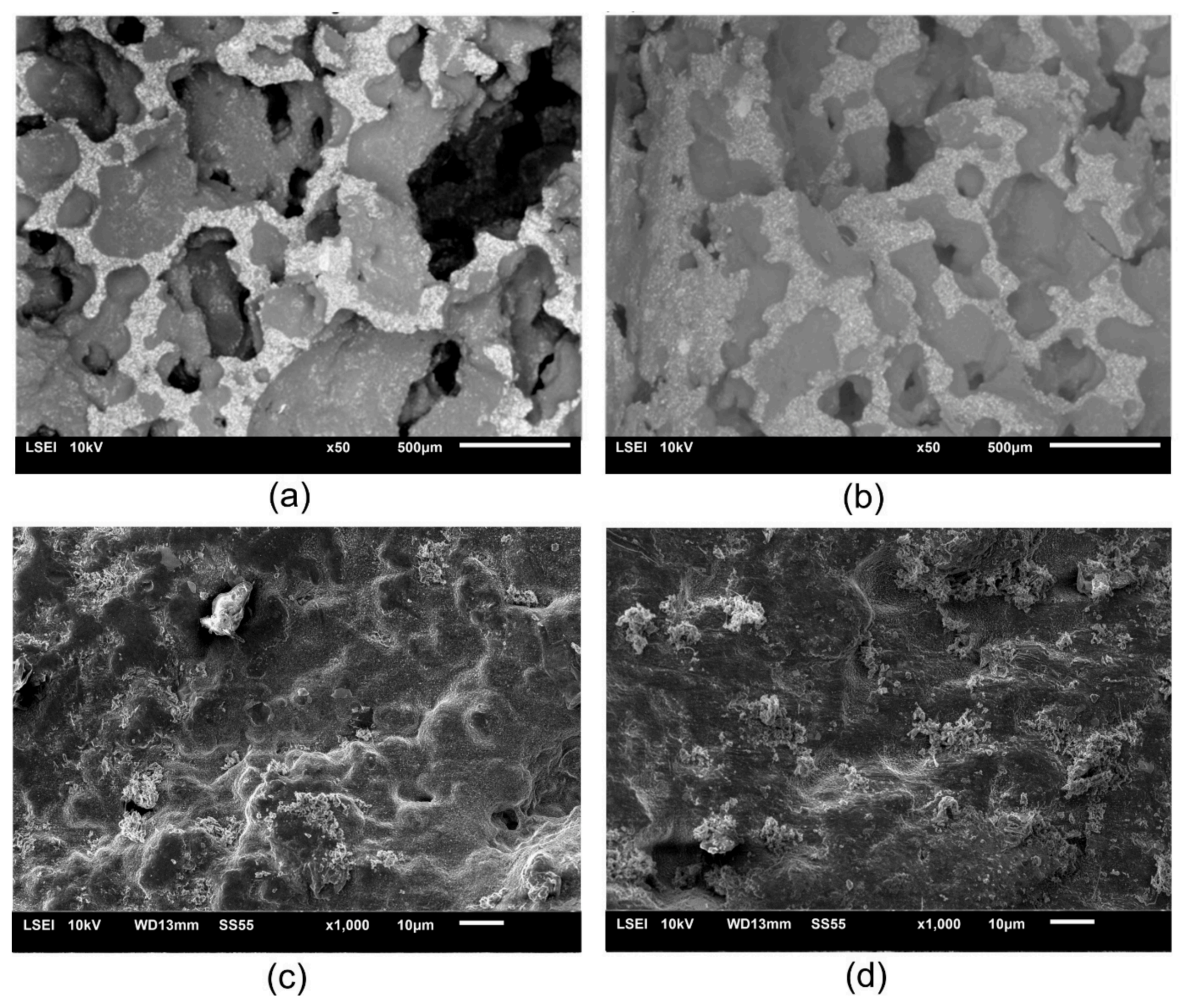
SEM micrographs of PCL-diol-based polyurethanes at ×500 magnification: (**a**) PU_3c_80 50×, (**b**) PU_3c_40 50×, (**c**) PU_3c_80 1000×, and (**d**) PU_3c_40 1000×.

**Figure 11:**
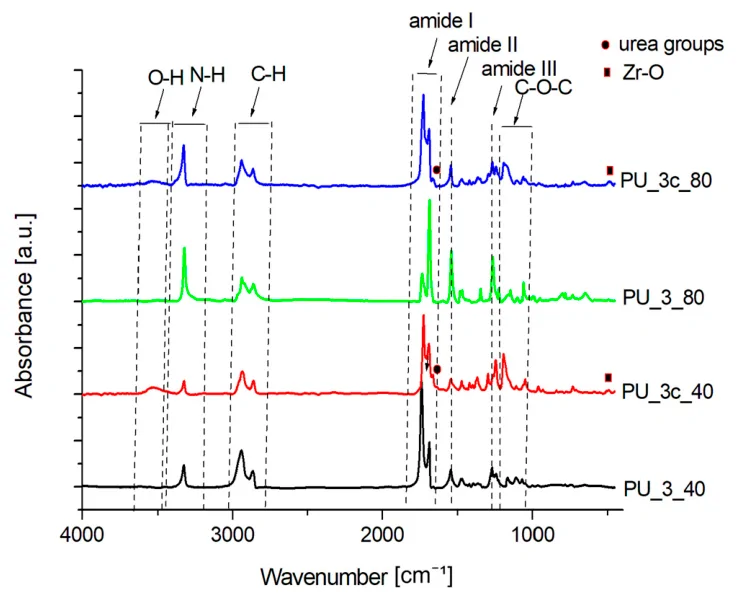
FTIR spectra obtained for samples PU_3_40, PU_3c_40, PU_3_80, and PU_3c_80.

**Table 1:** Synthesis parameters and molar ratios of synthesized polyurethanes.

Sample	Incubation Temperature (°C)	Molar Ratio HDI:PCL:BDO	Content of HS (%)	Content of Fillers (%)
PU_1_80	80	2:1:1	17.6	-
PU_2_80	80	2.5:1:1.5	21.7	-
PU_3_80	80	3:1:2	25.5	-
PU_3_40	40	3:1:2	25.5	-
PU_3c_80	80	3:1:2	25.5	20
PU_3c_40	40	3:1:2	25.5	20
PU_4_80	80	3.4:1:2.4	28.3	-
PU_5_80	80	4:1:3	32.0	-

**Table 2:** Positions of individual peaks of ester and amide bonds type I.

Sample	PCL Diol in the Polyurethane Structure	Polyurethane—Amide I		
	Stretching Vibrations of Ester C=O Groups	Stretching Vibrations of Free Urethane C=O Groups	Stretching Vibrations of Hydrogen-Bonded Urethane C=O Groups in Hard Domains	
PU_1_80	1720	1733	1695	-
PU_2_80	1719	1733	1692	-
PU_3_80	1719	1731	1684	-
PU_3_40	1716	1732	1687	-
PU_4_80	1717	1728	1685	1680
PU_5_80	1717	1728	1683	1671

**Table 3:** Values of the carbonyl hydrogen bonding index (R) and degree of phase separation (DPS).

Sample	Index R	Index DPS
PU_1_80	0.50	0.33
PU_2_80	0.58	0.37
PU_3_80	2.88	0.74
PU_3_40	2.62	0.72
PU_4_80	2.70	0.73
PU_5_80	3.10	0.76

**Table 4:** DSC test results of polyurethanes based on PCL diol.

Sample	Melting of Soft Segments		Melting of Hard Segments		X_c_ (%)
	T_m_ (°C)	ΔH_m_ (J/g)	T_m_ (°C)	ΔH_m_ (J/g)	
PU_1_80	42.6	54.2	121.7	16.0	48.4
PU_2_80	39.8	41.2	128.4	19.1	38.7
PU_3_80	37.0	38.1	130.4	22.2	36.2
PU_3_40	36.2	37.7	122.7	19.3	34.0
PU_4_80	34.3	25.4	140.5	26.2	26.0
PU_5_80	26.5	20.1	147.0	27.4	21.7

**Table 5:** Molecular weight characteristics of the selected polyurethane samples determined by GPC.

Sample	M_n_ (g/mol)	M_w_ (g/mol)	PDI
PU_3_40	44,300	80,620	1.819
PU_3_80	49,500	82,170	1.660

**Table 6:** Absorption bands of type I amide and ester bonds.

Sample	PCL Diol in the Polyurethane Structure	Polyurethane—Amide I		
	Stretching Vibrations of Ester C=O Groups	Stretching Vibrations of Free Urethane C=O Groups	Stretching Vibrations of Hydrogen-Bonded Urethane C=O Groups in Hard Domains	
PU 3 40	1716	1732	1687	-
PU3c 40	1711	1733	1684	-
PU3 80	1716	1731	1684	-
PU3c_80	1715	1733	1690	-

**Table 7:** Carbonyl hydrogen bonding index values and degree of phase separation (DPS).

Sample	Index R	Index DPS
PU_3_40	2.62	0.72
PU_3c_40	2.60	0.72
PU_3_80	2.88	0.74
PU_3c_80	1.58	0.61

**Table 8:** DSC test results of polyurethanes based on PCL diol.

Sample	Melting of Soft Segments		Melting of Hard Segments		Degree of Crystallinity of Soft Segments (%)
	T_m_ (°C)	ΔH_m_ (J/g)	T_m_ (°C)	ΔH_m_ (J/g)	
PU_3_40	36.2	37.7	122.7	19.3	34.0
PU_3c_40	35.3	37.0	126.3	20.1	33.1
PU_3_80	37.0	38.1	130.4	22.2	36.2
PU_3c_80	37.5	38.7	119.1	15.5	36.7

**Table 9:** Comparison of the results of solid and porous polyurethanes incubated at different temperatures.

Sample	Contact Angle Measurements			Mechanical Properties Analysis	Examination of Cell Cytotoxicity
	Polar Component (mJ/m^2^)	Dispersion Component (mJ/m^2^)	SFE (mJ/m^2^)	Young’s Modulus (MPa)	Average Viability of the Cells (%)
PU_3_40	2.4	38.3	40.7 ± 2.2	31.7 ± 2.7	99.8 ± 10.1
PU_3c_40	22.9	39.1	62.0 ± 2.6	29.5 ± 2.5	95.6 ± 6.8
PU_3_80	1.0	38.6	39.6 ± 3.1	36.9 ± 3.5	107.5 ± 13.4
PU_3c_80	13.3	42.2	55.5 ± 3.7	21.2 ± 3.0	86.5 ± 7.6

**Table 10:** Number-average molecular weight (M_n_), weight-average molecular weight (M_w_), polydispersity index (PDI), Young’s modulus, and mass loss of polyurethane samples before incubation and after 30 days of incubation at 37 °C.

Sample	M_n_ (g/mol)	M_w_ (g/mol)	PDI	Young’s Modulus (MPa)	∆m (%)
Samples before incubation					
PU_3_40	44,300	80,620	1.819	31.7 ± 2.7	-
PU_3c_40	-	-	-	29.5 ± 2.5	-
PU_3_80	49,500	82,170	1.660	36.9 ± 3.5	-
PU_3c_80	-	-	-	21.2 ± 3.0	-
Samples after 30 days of incubation					
PU_3_40	43,400	79,220	1.825	32.5 ± 2.8	0.60 ± 0.06
PU_3c_40	-	-	-	29.7 ± 2.1	0.63 ± 0.05
PU_3_80	49,110	81,550	1.661	36.0 ± 2.9	0.54 ± 0.03
PU_3c_80	-	-	-	22.0 ± 2.5	0.53 ± 0.04

## Data Availability

The original contributions presented in this study are included in the article. Further inquiries can be directed to the corresponding author.
